# Supplementary material for: Epigenetic reprogramming at estrogen-receptor binding sites alters 3D chromatin landscape in endocrine-resistant breast cancer
Source: Nat Commun. 2020 Jan 16;11:320. doi: 10.1038/s41467-019-14098-x (PMC6965612; doi:10.1038/s41467-019-14098-x)
Supplement: Supplementary file 2 — Supplementary Information [file 41467_2019_14098_MOESM2_ESM.pdf]

Epigenetic reprogramming at estrogen-receptor binding sites alters 3D chromatin landscape in endocrine resistant breast cancer

Achinger-Kawecka et al.

Supplementary Information

Supplementary Figure 1

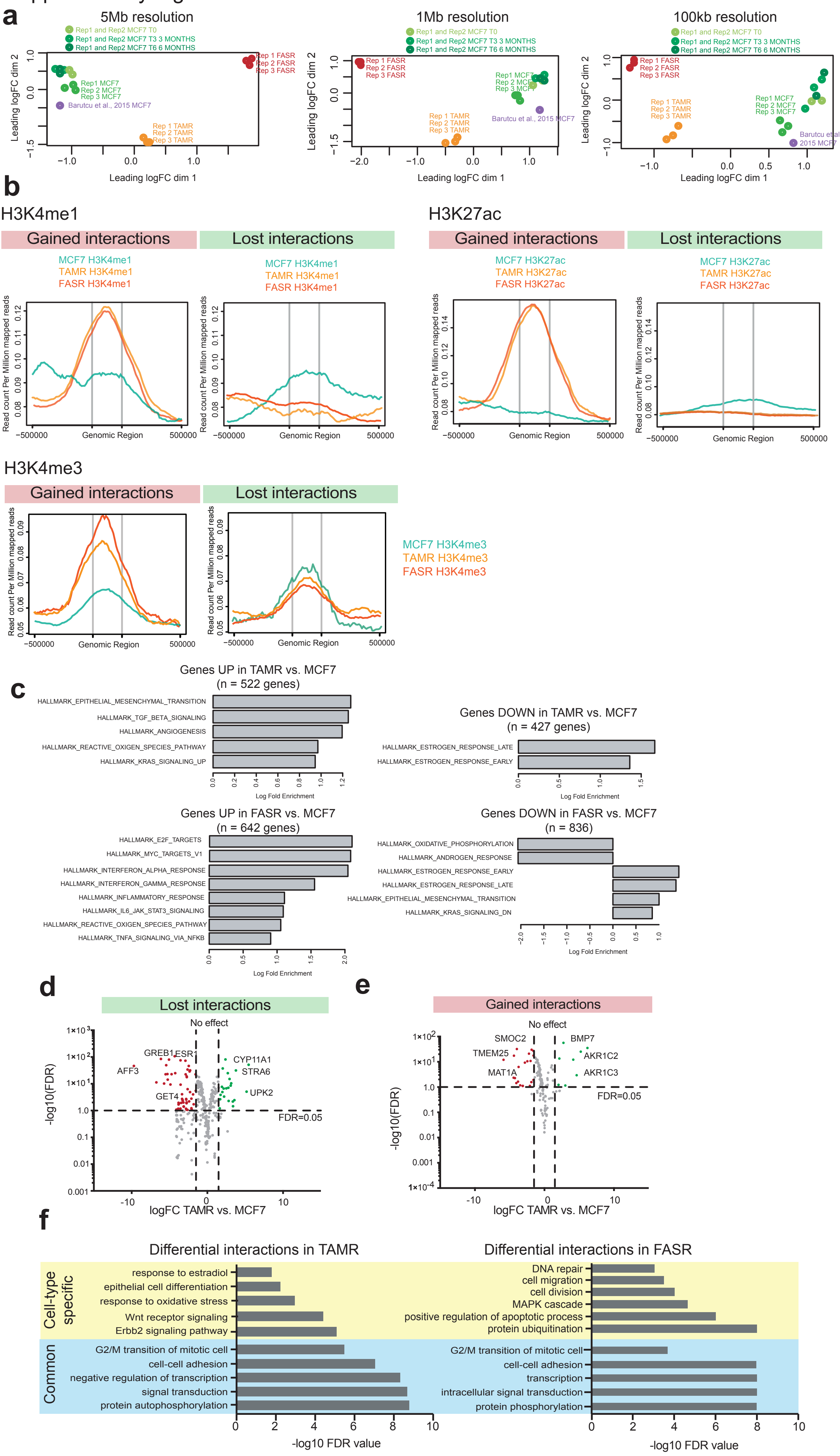

Supplementary Figure 1. Differential interactions in endocrine resistant breast cancer cells

a. MDS plot showing clustering between MCF7 (green; parental MCF7 cells, long-term culture MCF7 cells (T0, mid and 6months)), public MCF7 (purple) (Barutcu et al., 2015) and endocrine-resistant (orange and red) Hi-C samples based on top 1000 differential interactions at 5Mb, 1Mb and 100kb resolution.

b. Average plot of putative enhancer histone marks (H3K4me1 and H3K27ac) and putative promoter histone mark (H3K4me3) ChIP-seq signal in MCF7, TAMR and FASR cells at gained and lost differential interactions in TAMR and FASR cells.

c. Gene set enrichment analysis using MSigDB Hallmarks gene set for differentially expressed genes in TAMR (top panel) and FASR (bottom panel) as compared to MCF7 cells.

d. Volcano plot (-log10FDR vs. log2 Fold Change) of all genes present at anchors of lost differential interactions between TAMR and MCF7 cells. Source data are provided as a Source Data file.

e. Volcano plot (-log10FDR vs. log2 Fold Change) of all genes present at anchors of gained differential interactions between TAMR and MCF7 cells. Source data are provided as a Source Data file.

f. Gene Ontology Biological Processes terms enriched at differentially expressed genes (FDR < 0.05) located at anchors of differential interaction identified in TAMR (left panel) and FASR (right panel) cells. Terms specific to each resistant cell line are highlighted in yellow and terms enriched in both resistant cell lines are highlighted in blue.

Supplementary Figure 2

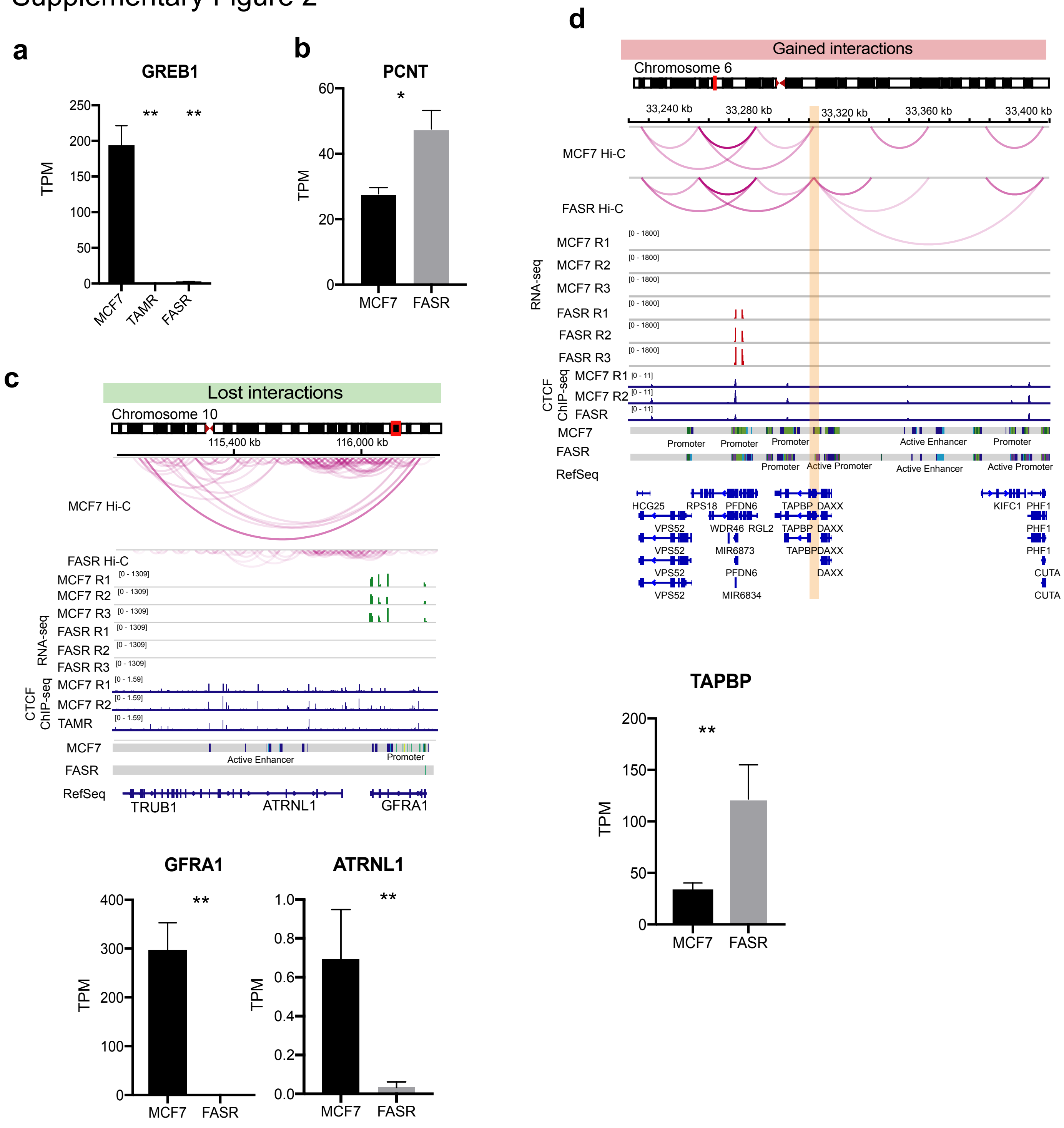

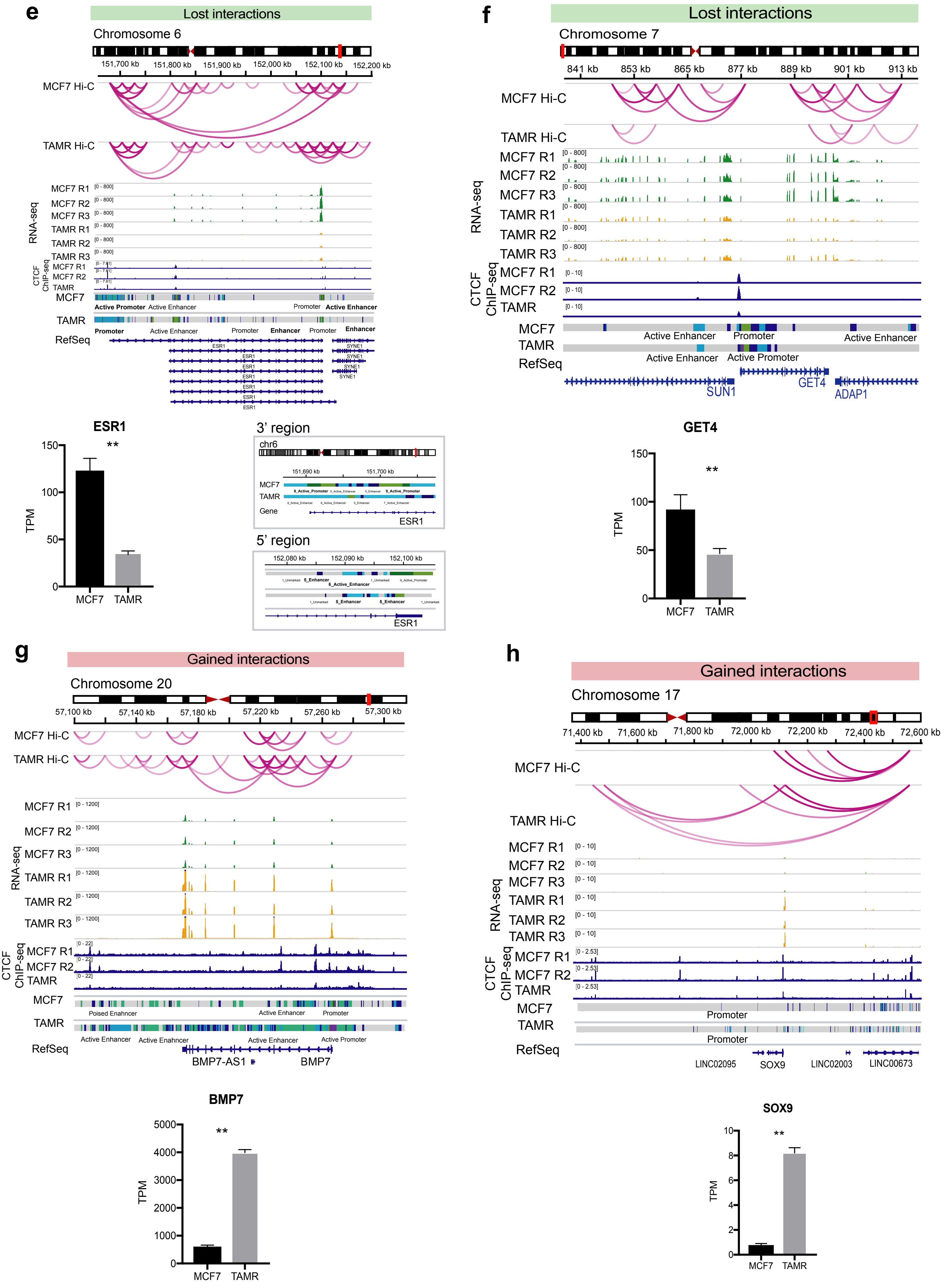
e
Lost interactions
Chromosome 6
151,700 kb
151,800 kb
151,900 kb
152,000 kb
152,100 kb
152,200 kb
MCF7 Hi-C
TAMR Hi-C
RNA-seq
MCF7 R1
MCF7 R2
MCF7 R3
TAMR R1
TAMR R2
TAMR R3
CTCF ChIP-seq
MCF7
TAMR
Active Promoter
Active Enhancer
Promoter
Enhancer
RefSeq
ESR1
SYNE1
ESR1
TPM
MCF7
TAMR
**
3' region
chr6
151,690 kb
151,700 kb
Gene
5' region
152,080 kb
152,090 kb
152,100 kb
f
Lost interactions
Chromosome 7
841 kb
853 kb
865 kb
877 kb
889 kb
901 kb
913 kb
SUN1
GET4
ADAP1
GET4
g
Gained interactions
Chromosome 20
57,100 kb
57,140 kb
57,180 kb
57,220 kb
57,260 kb
57,300 kb
Poised Enhancer
BMP7-AS1
BMP7
BMP7
h
Gained interactions
Chromosome 17
71,400 kb
71,600 kb
71,800 kb
72,000 kb
72,200 kb
72,400 kb
72,600 kb
LINC02095
SOX9
LINC02003
LINC00673
SOX9

Supplementary Figure 2. Differential interactions associate with gene deregulation

a. *GREB1* mRNA expression in lost in TAMR cells and FASR cells (**P value < 0.001)

b. *PCNT1* mRNA expression in up-regulated in FASR cells (*P value < 0.05)

c. Representative example demonstrating the association between enhancer-promoter interactions lost in FASR cells as compared to MCF7 cells and decreased expression of *GFRA1* and *ATRNL1* genes. *GFRA1* and *ATRNL1* mRNA expression in down-regulated in FASR cells (**P value < 0.001)

d. Representative example demonstrating the association between enhancer-promoter interactions gained in FASR cells as compared to MCF7 cells and overexpression of *TAPBP* gene. *TAPBP* mRNA expression in up-regulated in FASR cells (**P value < 0.001)

e. Representative example demonstrating the association between enhancer-promoter interactions lost in TAMR cells as compared to MCF7 cells and decreased expression of *ESR1* gene. Zoomed-in views of chromHMM segmentation 3' (promoter) and 5' (enhancer) region of *ESR1* gene are shown. *ESR1* mRNA expression in down-regulated in TAMR cells (**P value < 0.001)

f. Representative example demonstrating the association between enhancer-promoter interactions lost in TAMR cells as compared to MCF7 cells and decreased expression of *GET4* gene. *GET4* mRNA expression in down-regulated in TAMR cells (**P value < 0.001)

g. Representative example demonstrating the association between enhancer-promoter interactions gained in TAMR cells as compared to MCF7 cells and overexpression of *BMP7* gene. *BMP7* mRNA expression in up-regulated in TAMR cells (**P value < 0.001)

h. Representative example demonstrating the association between enhancer-promoter interactions gained in TAMR cells as compared to MCF7 cells and overexpression of *SOX9* gene. *SOX9* mRNA expression in up-regulated in TAMR cells (**P value < 0.001)

Supplementary Figure 3

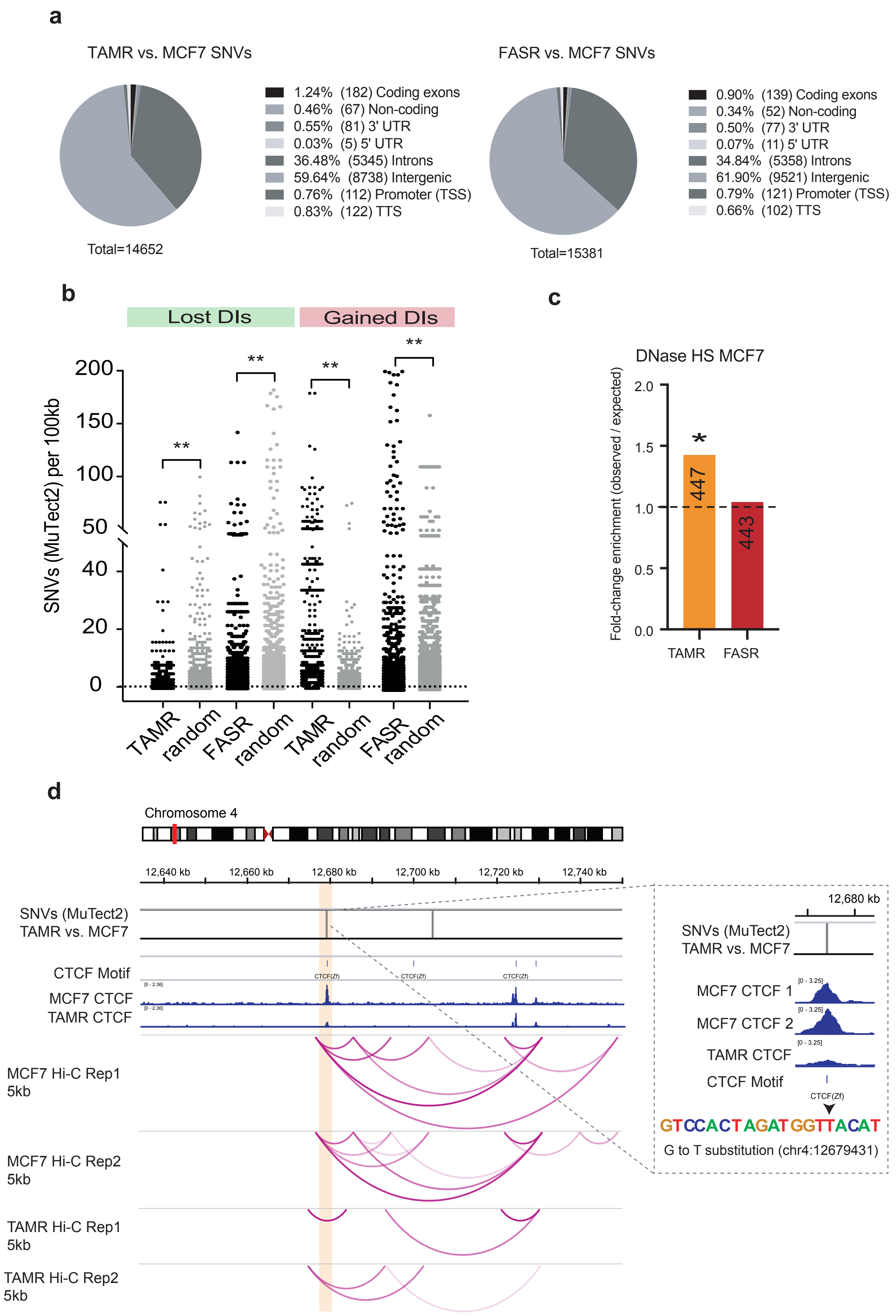

e

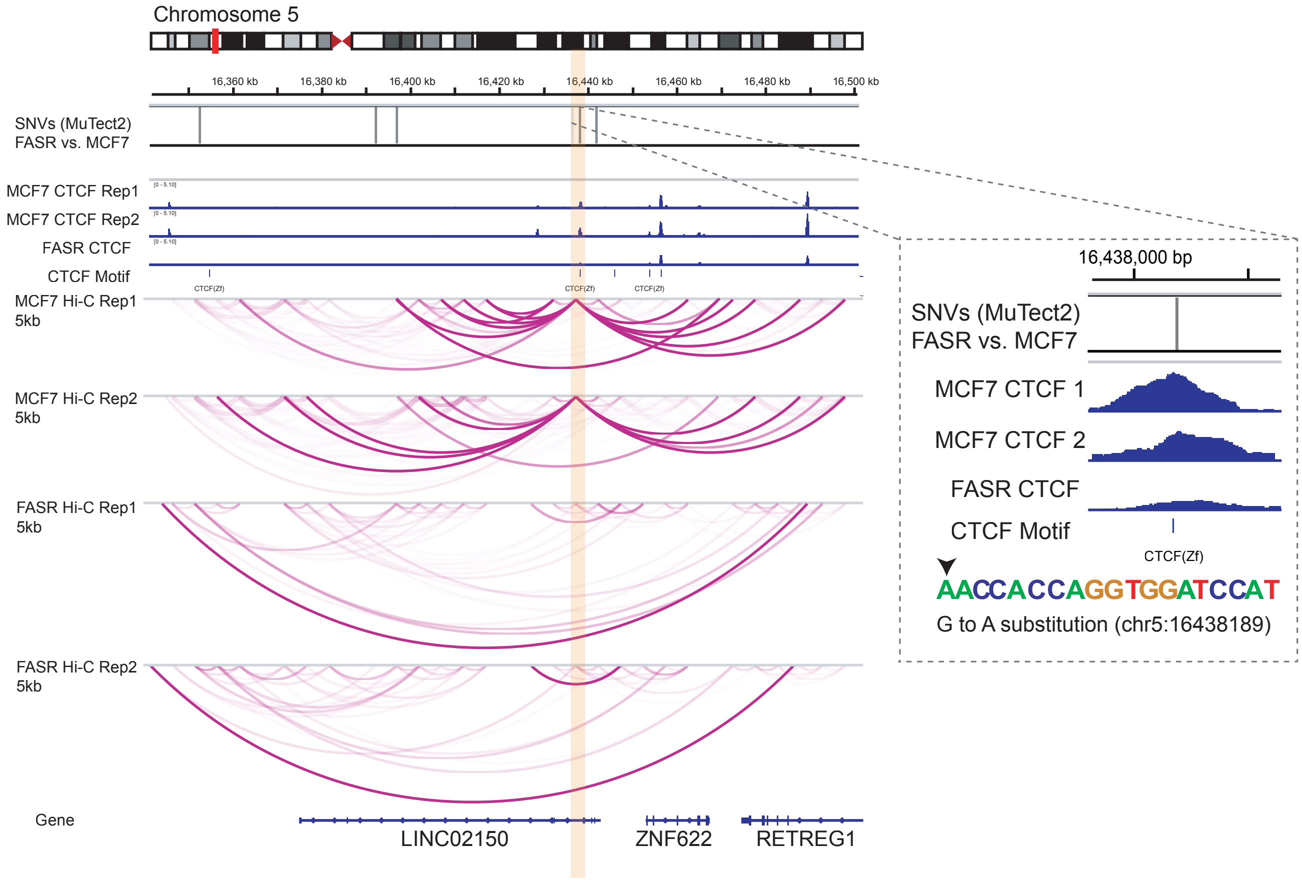
Chromosome 5
16,360 kb
16,380 kb
16,400 kb
16,420 kb
16,440 kb
16,460 kb
16,480 kb
16,500 kb
SNVs (MuTect2)
FASR vs. MCF7
MCF7 CTCF Rep1
MCF7 CTCF Rep2
FASR CTCF
CTCF Motif
CTCF(Zf)
MCF7 Hi-C Rep1
5kb
MCF7 Hi-C Rep2
5kb
FASR Hi-C Rep1
5kb
FASR Hi-C Rep2
5kb
Gene
LINC02150
ZNF622
RETREG1
16,438,000 bp
SNVs (MuTect2)
FASR vs. MCF7
MCF7 CTCF 1
MCF7 CTCF 2
FASR CTCF
CTCF Motif
CTCF(Zf)
AACCACCAGGTGGATCCAT
G to A substitution (chr5:16438189)

f

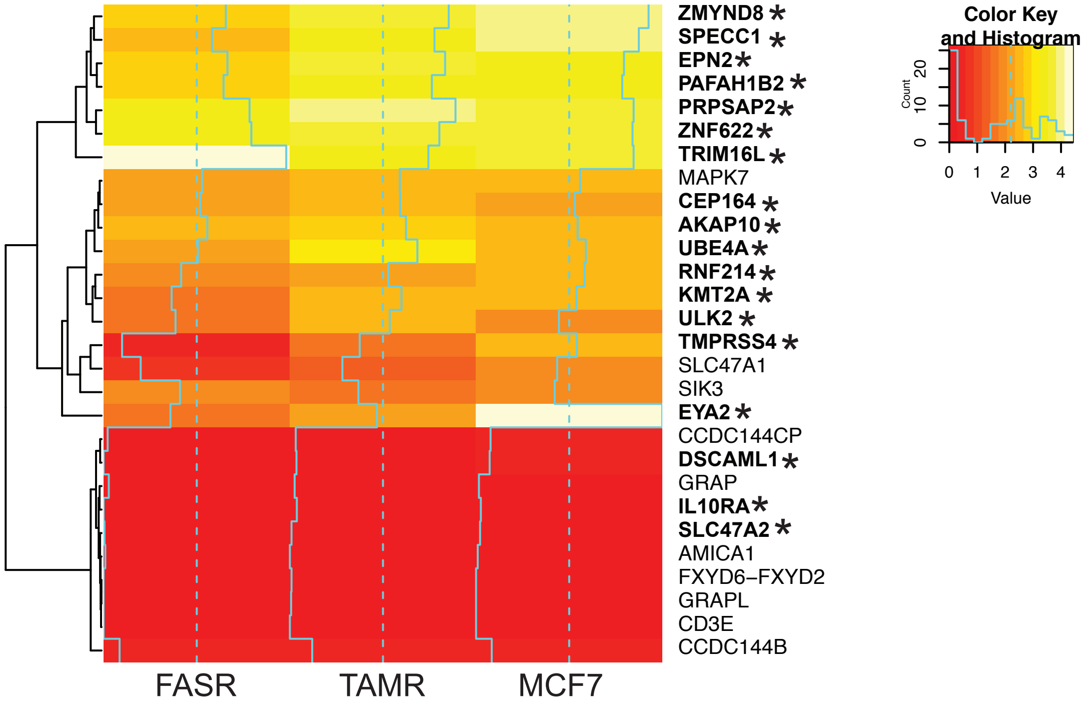
ZMYND8 *
SPECC1 *
EPN2 *
PAFAH1B2 *
PRPSAP2 *
ZNF622 *
TRIM16L *
MAPK7
CEP164 *
AKAP10 *
UBE4A *
RNF214 *
KMT2A *
ULK2 *
TMPRSS4 *
SLC47A1
SIK3
EYA2 *
CCDC144CP
DSCAML1 *
GRAP
IL10RA *
SLC47A2 *
AMICA1
FXYD6–FXYD2
GRAPL
CD3E
CCDC144B
FASR
TAMR
MCF7
Color Key
and Histogram
Count
0
10
20
0
1
2
3
4
Value

Supplementary Figure 3. SNVs associate with loss of interactions and gene deregulation

a. Annotation of identified endocrine-resistance associated SNVs to genomic regions from Homer annotatePeaks.py

b. The number of single nucleotide variants per 100kb region that are located at lost and gained differential interactions in TAMR and FASR cells. Asterisks represent the significance of overlap at observed versus randomized DIs (permutation test ** P value < 0.0001; * P value < 0.05)

c. Endocrine-resistance associated SNVs enrichment for MCF7 DNase-Seq HS sites. DNase-Seq data obtained from ENCODE.

d. MCF7 (top panel) and TAMR (bottom panel) Hi-C interactions map on chromosome 4 showing a differential interaction, which is lost in TAMR cells and associated with a resistance-specific SNV (G to A substitution) located within a CTCF binding site at CTCF motif (highlighted in orange). In the zoom-in view of the SNV, CTCF ChIP-seq tracks, SNV flanking sequence (bottom) and location at the CTCF motif and lost CTCF binding are shown.

e. MCF7 (top panel) and FASR (bottom panel) Hi-C interactions map on chromosome 5 showing a differential interaction, which is lost in FASR cells and associated with a resistance-specific SNV (G to A substitution) located within a CTCF binding site at CTCF motif (highlighted in orange). In the zoom-in view of the SNV, CTCF ChIP-seq tracks, SNV flanking sequence (bottom) and location at the CTCF motif and lost CTCF binding are shown.

f. Expression (logTPM) of genes, which promoters overlap differential interactions associated with endocrine resistance-associated SNVs located at lost CTCF binding sites. Differentially expressed genes either in TAMR vs. MCF7 or FASR vs. MCF7 are marked by asterisk (FDR < 5%).

Supplementary Figure 4

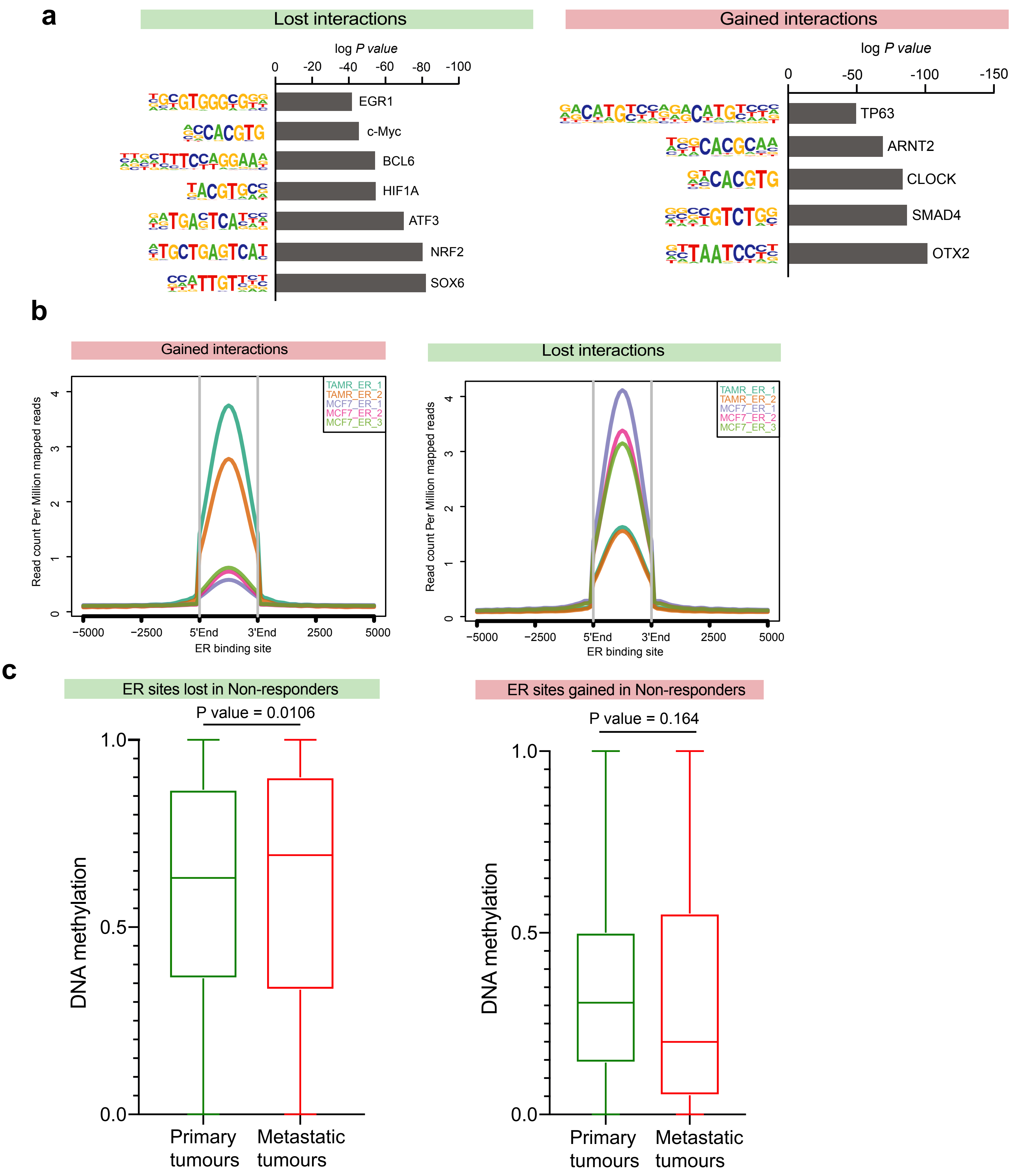

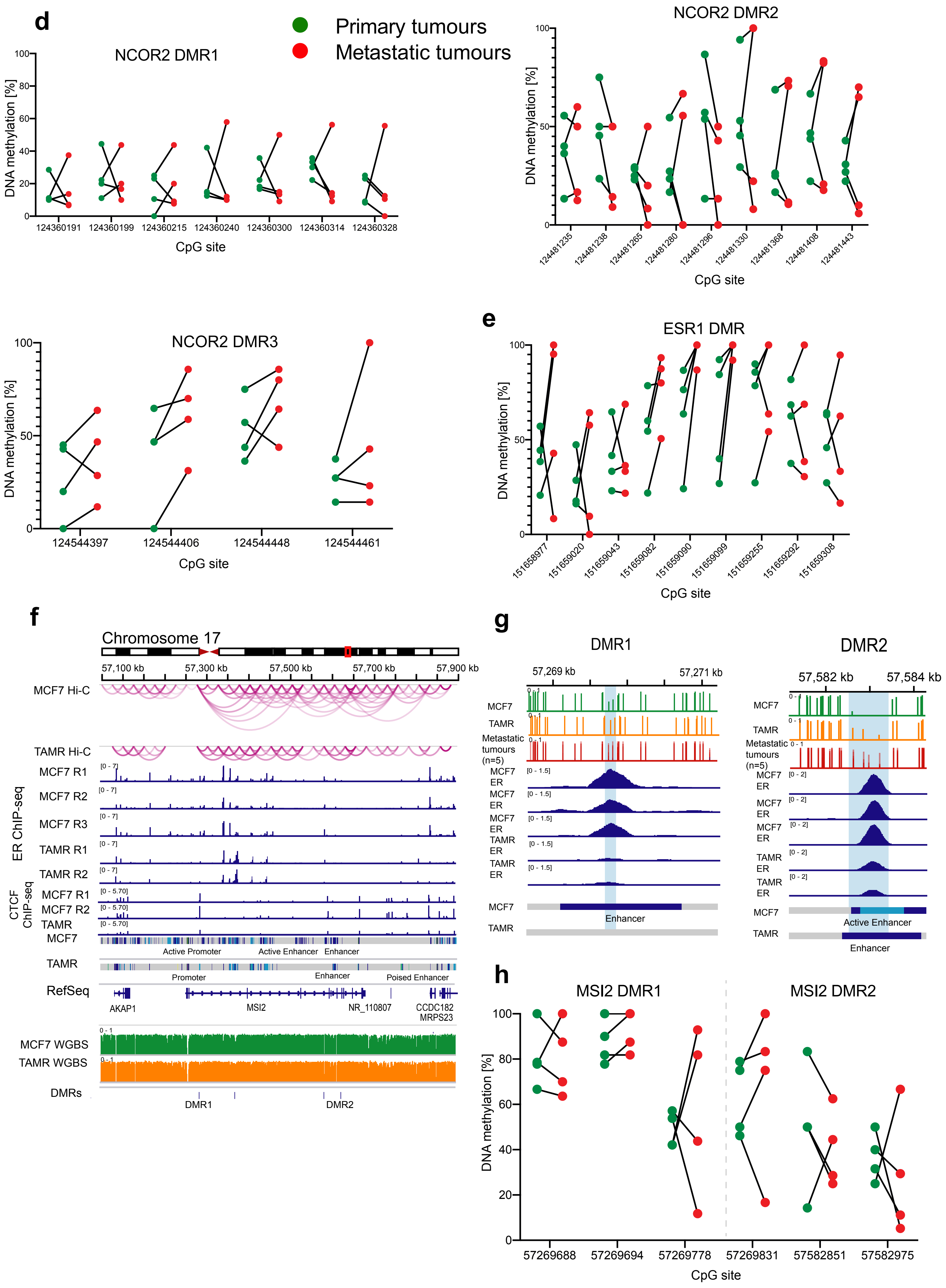

d
Primary tumours
Metastatic tumours
NCOR2 DMR1
DNA methylation [%]
CpG site
124360191
124360199
124360215
124360240
124360300
124360314
124360328
NCOR2 DMR2
124481235
124481238
124481265
124481280
124481296
124481330
124481368
124481408
124481443
NCOR2 DMR3
124544397
124544406
124544448
124544461
e
ESR1 DMR
151658977
151659020
151659043
151659082
151659090
151659099
151659255
151659292
151659308
f
Chromosome 17
57,100 kb
57,300 kb
57,500 kb
57,700 kb
57,900 kb
MCF7 Hi-C
TAMR Hi-C
ER ChIP-seq
MCF7 R1
MCF7 R2
MCF7 R3
TAMR R1
TAMR R2
CTCF ChIP-seq
TAMR
MCF7
Active Promoter
Active Enhancer
Enhancer
Promoter
Poised Enhancer
RefSeq
AKAP1
MSI2
NR_110807
CCDC182
MRPS23
MCF7 WGBS
TAMR WGBS
DMRs
DMR1
DMR2
g
DMR1
57,269 kb
57,271 kb
DMR2
57,582 kb
57,584 kb
Metastatic tumours (n=5)
MCF7 ER
TAMR ER
h
MSI2 DMR1
MSI2 DMR2
57269688
57269694
57269778
57269831
57582851
57582975

Supplementary Figure 4. Differential interactions occur at regions of DNA hypermethylation

a. Motifs enriched at anchors of lost (left panel) and gained (right panel) interactions between FASR and MCF7 cells. Known motifs obtained from Homer database (hg38) and compared to matched, randomized background regions.

b. Average profile of ER ChIP-seq signal at ER binding sites located at gained and lost interactions in TAMR cells.

c. (Left panel) Gain in DNA methylation in metastatic tumours (n = 5) at ER binding sites lost on Non-responders as compared to DNA methylation levels in primary tumours (n = 4). (Right panel) Loss of DNA methylation in metastatic tumours (n = 5) at ER binding sites gained in Non-responders as compared to DNA methylation levels in primary tumours (n = 4). ER ChIP-seq data obtained from (Ross-Innes et al., Nature 2012). Source data are provided as a Source Data file.

d. Percentage of DNA methylation at each CpG site within *NCOR2* DMRs in primary tumours (n = 4) (shown in green) and matched metastatic tumours (n = 4) (shown in red). Lines represent matching between samples. Source data are provided as a Source Data file.

e. Percentage of DNA methylation at each CpG site within the *ESR1* DMR in primary tumours (n = 4) (shown in green) and matched metastatic tumours (n = 4) (shown in red). Lines represent matching between samples. Source data are provided as a Source Data file.

f. Representative example showing loss of ER-bound interaction in TAMR cells at *MSI2* gene associated with DNA hypermethylation. Two differentially methylated regions (DMRs) that are present at ER-enhancer regions are associated with loss of ER binding and loss of interactions in TAMR cells.

g. Differentially methylated regions associated with loss of ER-bound interactions in TAMR cells. DNA hypermethylation at the region of ER binding in MCF7 cells can be observed in metastatic ER+ breast cancer patient tumour samples (n = 5) as compared to primary tumours (n = 4).

h. Percentage of DNA methylation at each CpG site within *MSI2* DMRs in primary tumours (n = 4) (shown in green) and matched metastatic tumours (n = 4) (shown in red). Lines represent matching between samples. Source data are provided as a Source Data file.

Supplementary Figure 5

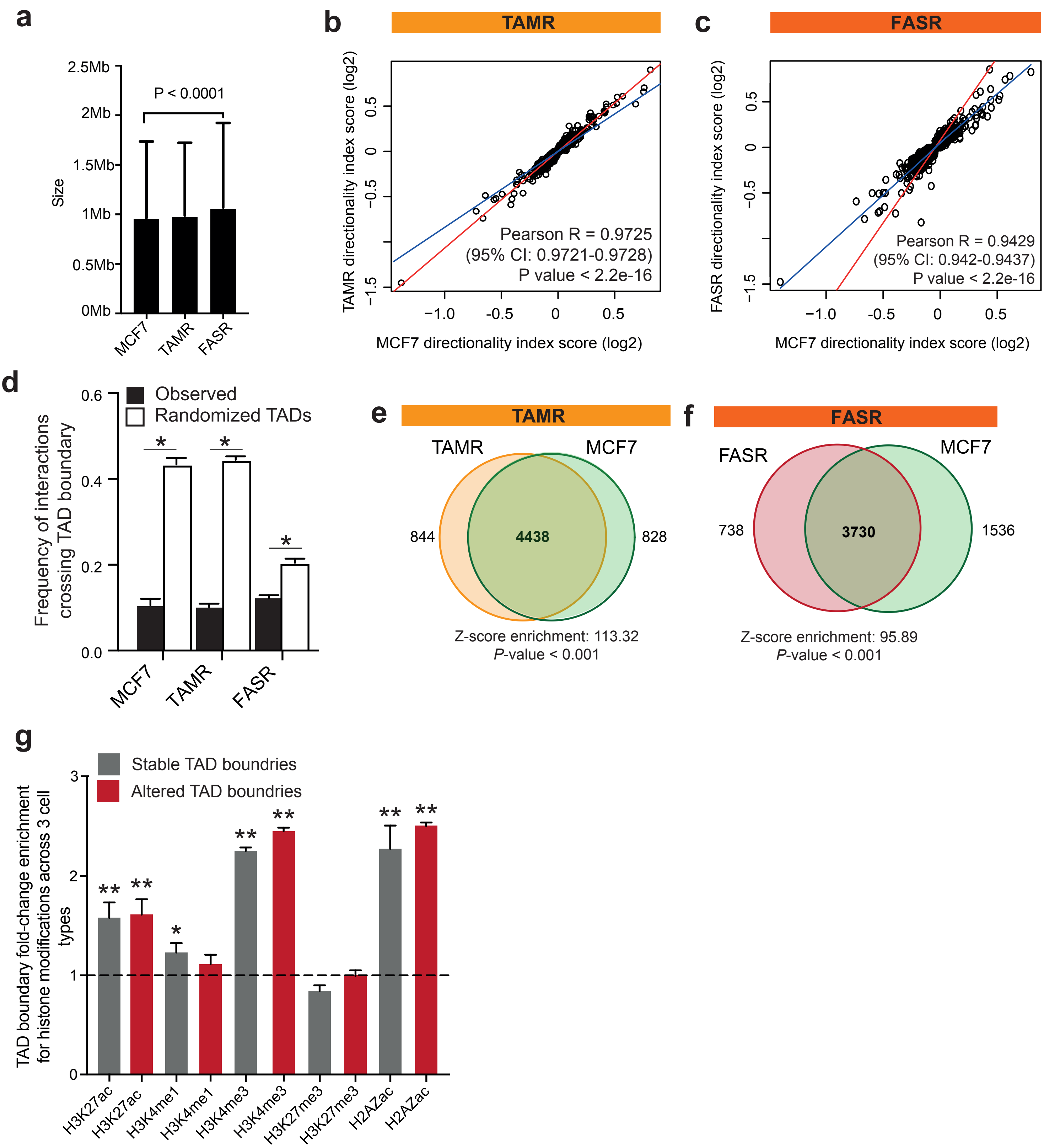

h

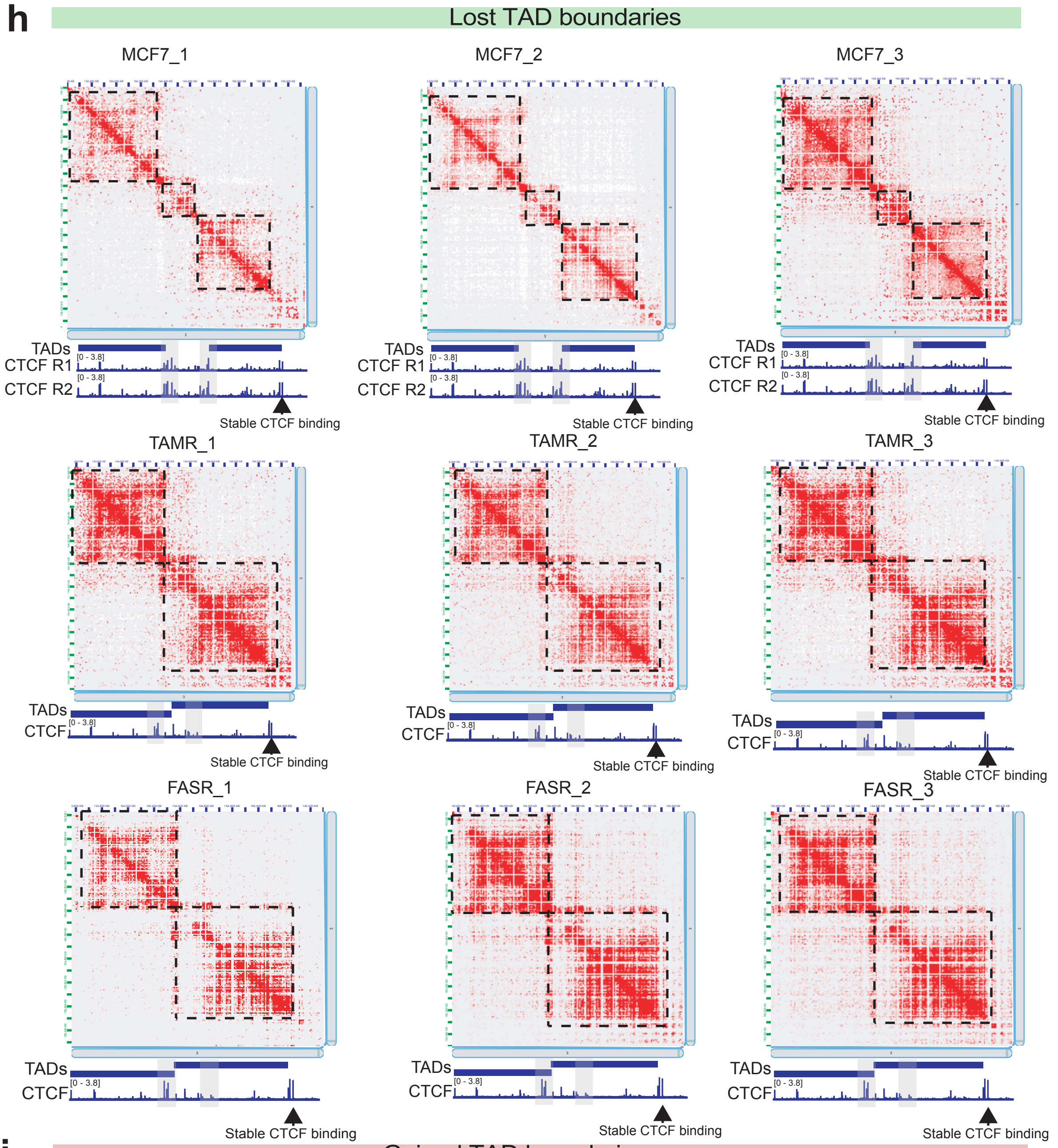
Lost TAD boundaries
MCF7_1
MCF7_2
MCF7_3
TADs
CTCF R1
CTCF R2
[0 - 3.8]
Stable CTCF binding
TAMR_1
TAMR_2
TAMR_3
TADs
CTCF
[0 - 3.8]
Stable CTCF binding
FASR_1
FASR_2
FASR_3
TADs
CTCF
[0 - 3.8]
Stable CTCF binding

i

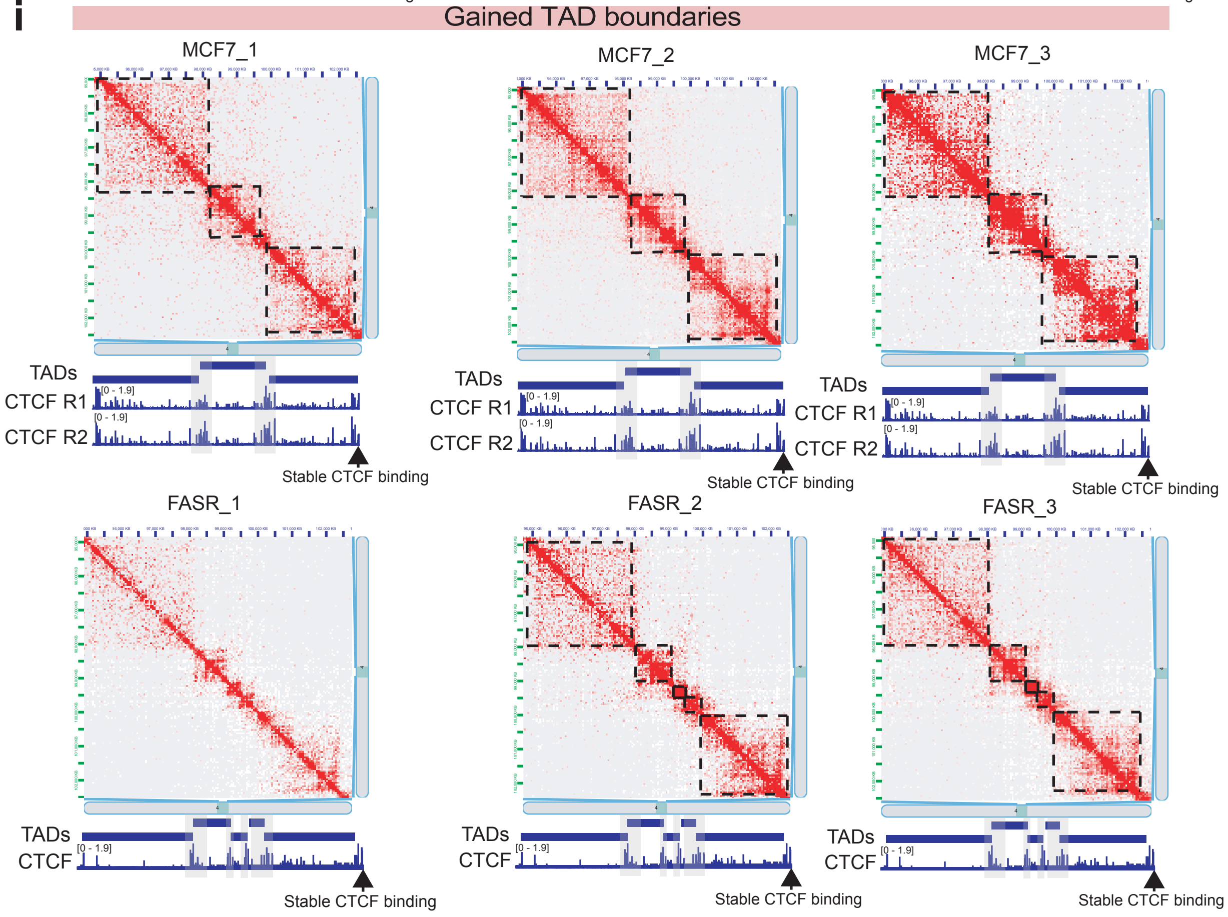
Gained TAD boundaries
MCF7_1
MCF7_2
MCF7_3
TADs
CTCF R1
CTCF R2
[0 - 1.9]
Stable CTCF binding
FASR_1
FASR_2
FASR_3
TADs
CTCF
[0 - 1.9]
Stable CTCF binding

Supplementary Figure 5. Altered TAD boundaries in endocrine resistant breast cancer cells

a. TAD size in MCF7, TAMR and FASR cells. P value Student's t-test. Source data are provided as a Source Data file.

b. Pearson correlation between TAD boundary directionality index score (log2) in MCF7 and TAMR cells. Linear regression line shown in red, LOWESS regression line shown in blue.

c. Pearson correlation between TAD boundary directionality index score (log2) in MCF7 and FASR cells. Linear regression line shown in red, LOWESS regression line shown in blue.

d. Observed frequency of interactions (*cis* only) crossing TAD boundaries in each cell type compared to expected frequencies of TAD boundary-crossing interactions. Error bars show SD across 100 permutations (Student's t-test * P value < 0.0001).

e. Numbers of unique and overlapping TAD boundaries in TAMR and MCF7 cells. Z-score of significance of overlap compared to random, distance-matched regions.

f. Numbers of unique and overlapping TAD boundaries in FASR and MCF7 cells. Z-score of significance of overlap compared to random, distance-matched regions.

g. Histone modification enrichment at TAD boundaries, compared to random, distance-matched regions in three cell types. Asterisks represent the significance of enrichment at observed versus random regions (permutation test ** P value < 0.001; * P value < 0.05). Source data are provided as a Source Data file.

h. Per Hi-C replicate data (n = 3) showing loss of TAD boundaries commonly observed in TAMR and FASR cells. Interaction heatmaps in JuiceBox aligned with CTCF ChIP-seq showing segmentation into TADs. Arrow marks a TAD boundary present in MCF7 cells and marked by high CTCF binding, which is lost in TAMR and FASR cells.

i. Per Hi-C replicate data (n = 3) showing gain of TAD boundaries in FASR cells. Interaction heatmaps in JuiceBox aligned with CTCF ChIP-seq showing segmentation into TADs. Arrow marks an ectopic TAD boundary present in FASR cells and marked by high CTCF binding, which is lost in MCF7 cells.

Supplementary Figure 6

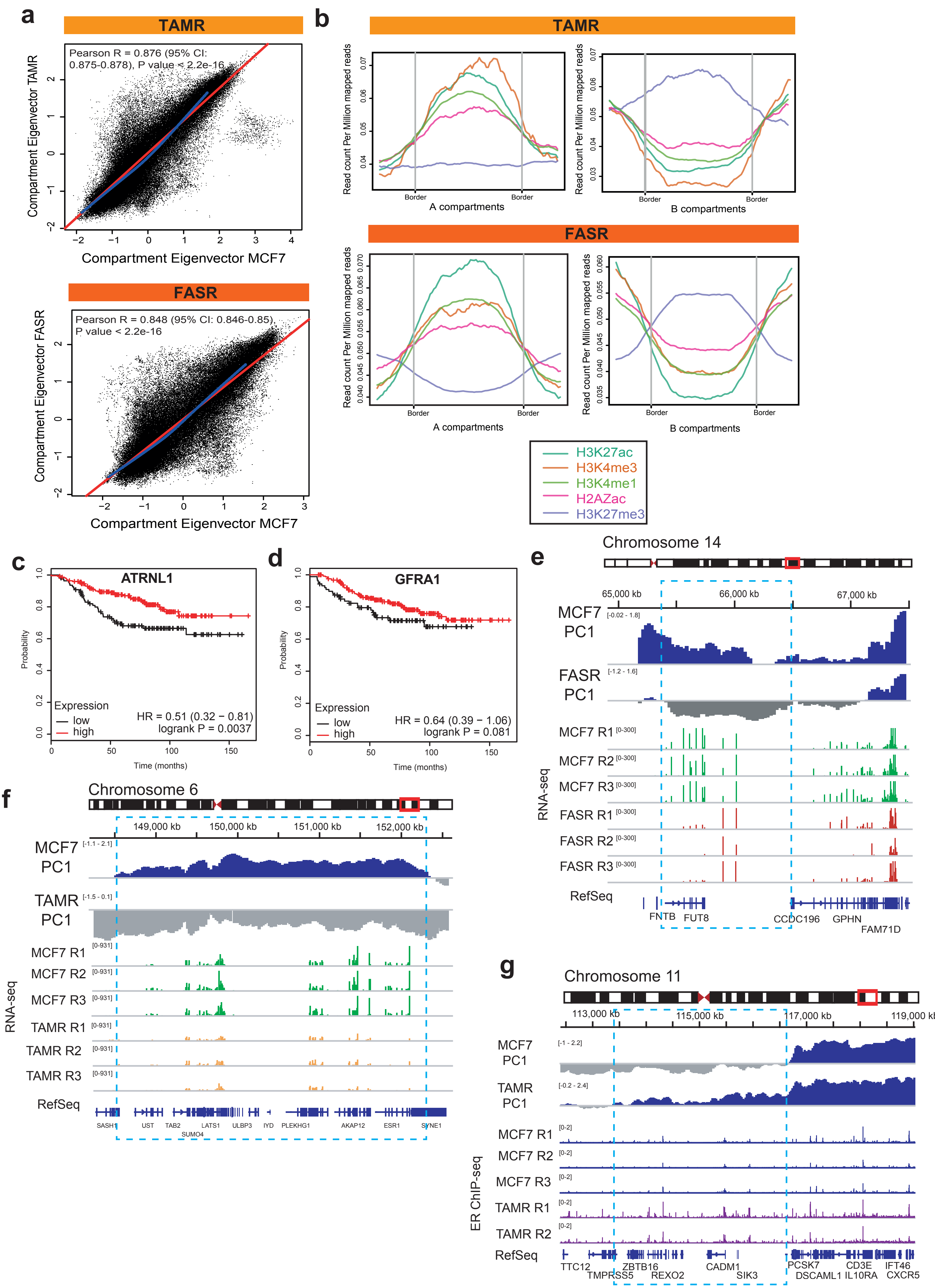

Supplementary Figure 6. Altered A/B compartment structure in endocrine resistant breast cancer cells

a. Scatter plot showing the degree of correlation (Pearson's) between eigenvector values estimated by eigenvector analysis of MCF7 and TAMR (top) and MCF7 and FASR (bottom) normalized Hi-C contact matrixes. Linear regression line shown in red, LOWESS regression line shown in blue.

b. Average histone modification profiles (H3K27ac, H3K4me3, H3K4me1, H2Azac and H3K27me3) over A and B compartments in TAMR and FASR cells.

c. Kaplan-Meier curves displaying relapse-free survival for 742 patients with ER+ tumours receiving endocrine treatment based on *ATRNL1* gene expression. Patients with tumors with high expression of *ATRNL1* are shown in red and those with low expression are shown in black. P value as indicated, log rank test.

d. Kaplan-Meier curves displaying relapse-free survival for 742 patients with ER+ tumours receiving endocrine treatment based on *GFRA1* gene expression. Patients with tumours with high expression of *GFRA1* are shown in red and those with low expression are shown in black. P value as indicated, log rank test.

e. An example of a region on chromosome 14 showing the compartment switching from "B-type" in parental MCF7 cells to "A-type" in endocrine-resistant FASR cells that is associated with loss of expression of *FUT8* gene in FASR cells as compared to MCF7 cells.

f. An example of a region on chromosome 6 showing the compartment switching from A-type in parental MCF7 cells to B-type in endocrine-resistant TAMR cells that is associated with loss of expression of multiple genes: *LATS1, ULBP3, PLEKHG1, AKAP12, ESR1* and *SYNE1* in TAMR cells as compared to MCF7 cells.

g. An example of a region on chromosome 11 showing the compartment switching from "B-type" in parental MCF7 cells to "A-type" in endocrine-resistant TAMR cells (marked in grey) that is associated with gain of ER binding in TAMR cells as compared to MCF7 cells

Supplementary Tables

Dataset	Total paired-end reads (ditags)	Uniquely mapped ditags	Valid interactions	Cis interactions
MCF7_1	65,126,894	34,611,823	31,497,834	22,642,261
MCF7_2	243,824,930	134,689,550	118,067,314	80,766,123
MCF7_3	215,222,684	116,024,362	100,450,982	68,799,133
Total MCF7	524,174,508	285,325,735 (54.4%)	243,028,717 (85.2%)	172,207,517 (70.9%)
MCF7_1 T0	62,281,884	30,168,711	26,166,521	18,700,506
MCF7_2 T0	59,234,419	21,344,987	17,884,324	11,963,969
MCF7_1 T3	53,553,294	30,678,069	27,302,671	20,494,594
MCF7_2 T3	50,618,470	24,588,983	21,496,060	15,724,230
MCF7_1 T6	59,510,721	29,192,912	25,797,973	18,989,861
MCF7_2 T6	49,028,640	14,338,357	10,101,529	69,31113
TAMR_1	137,194,260	76,921,539	64,532,994	48,098,206
TAMR_2	165,501,724	80,407,216	70,121,014	47,723,895
TAMR_3	244,853,950	108.517,341	96,734,779	59,848,264
Total TAMR	547,549,934	265,846,096 (48.5%)	226,253,736 (85.1%)	155,670,365 (68.8%)
FASR_1	122,471,276	54,414,546	47,032,634	30,990,656
FASR_2	142,234,628	81,224,765	70,771,784	53,841,253
FASR_3	126,412,318	78,949,189	66,383,987	49,402,640
Total FASR	391,118,222	214,588,500 (54.8%)	177,708,714 (82.8%)	134,234,549 (75.5%)
**SUM**	**1,462,842,664**	**765,760,331**	**646,991,167**	**462,112,431**

Supplementary Table 1

Hi-C library sequencing information for each replicate used in the study. Percentage of double-stranded uniquely mapped and filtered pairs indicates percentage to all processed pairs, and percentage of valid interactions indicates percentage of valid pairs to double-stranded uniquely mapped pairs. Cis ratio indicated ratio of *cis* (intra-chromosomal) interactions to all valid interactions.

	Filter Instances	
	TAMR vs. MCF7	FASR vs. MCF7
Alt allele in normal	43421	53065
Germline risk	80935	80000
Clustered event	53345	72075
Homologous mapping event	46	77
Multi event	79	70
Str contraction	968	1559
T_lod_fstar	14180	18101
Triallelic site	8	20
Filtered calls	192982	224967
**PASS calls**	**14652**	**15381**
**Common to TAMR and FASR**	**2254**	**2254**
Total calls	207634	240348

Supplementary Table 2
SNVs identified by Mutect2 in TAMR vs. MCF7 and FASR vs. MCF7 analyses.

Dataset	Accession Number	Reference
Hi-C MCF7	GSE118712	This paper
Hi-C TAMR	GSE118712	This paper
Hi-C FASR	GSE118712	This paper
Hi-C MCF7	GSE66733	Barutcu et al., Genome Biol 2015
RNA-seq MCF7	GSE118713	This paper
RNA-seq TAMR	GSE118713	This paper
RNA-seq FASR	GSE118713	This paper
WGS MCF7	GSE118715	This paper
WGS TAMR	GSE118715	This paper
WGS FASR	GSE118715	This paper
WGBS MCF7	GSE118714	This paper
WGBS TAMR	GSE118714	This paper
WGBS FASR	GSE118714	This paper
CTCF ChIP-seq MCF7	ENCODE	ENCSR000DWH
CTCF ChIP-seq TAMR	GSE118711	This paper
CTCF ChIP-seq FASR	GSE118711	This paper
H3K4me3 ChIP-seq MCF7	GSE57498	Taberlay et al., Genome Res 2014
H3K4me3 ChIP-seq TAMR	GSE118711	This paper
H3K4me3 ChIP-seq FASR	GSE118711	This paper
H3K4me1 ChIP-seq MCF7	GSE57498	Taberlay et al., Genome Res 2014
H3K4me1 ChIP-seq TAMR	GSE118711	This paper

H3K4me1 ChIP-seq FASR	GSE118711	This paper
H3K27ac ChIP-seq MCF7	GSE57498	Taberlay et al., Genome Res 2014
H3K27ac ChIP-seq TAMR	GSE118711	This paper
H3K27ac ChIP-seq FASR	GSE118711	This paper
H3K27me3 ChIP-seq MCF7	GSE57498	Taberlay et al., Genome Res 2014
H3K27me3 ChIP-seq TAMR	GSE118711	This paper
H3K27me3 ChIP-seq TAMR	GSE118711	This paper
H2AZac ChIP-seq MCF7	GSE118711	This paper
H2Azac ChIP-seq TAMR	GSE118711	This paper
H2Azac ChIP-seq FASR	GSE118711	This paper
chromHMM MCF7	GSE118711	This paper
chromHMM TAMR	GSE118711	This paper
chromHMM FASR	GSE118711	This paper
ER ChIP-seq MCF7	GSE32222	Ross-Innes et al., Nature 2012
ER ChIP-seq TAMR	GSE32222	Ross-Innes et al., Nature 2012
ER ChIP-seq Clinical ER+ breast cancer samples	GSE32222	Ross-Innes et al., Nature 2012
ReMap 2018 v1.2 MCF7		Cheneby et al., Nucl Acid Res 2018
MCF7 DNase-Seq	ENCSR000EPJ	

Supplementary Table 3

Datasets used in the study.
