# Supplementary material for: Epigenetic reprogramming at estrogen-receptor binding sites alters 3D chromatin landscape in endocrine-resistant breast cancer
Source: Nat Commun. 2020 Jan 16;11:320. doi: 10.1038/s41467-019-14098-x (PMC6965612; doi:10.1038/s41467-019-14098-x)
Supplement: Supplementary file 3 — Description of Additional Supplementary Files [file 41467_2019_14098_MOESM3_ESM.pdf]

Description of Additional Supplementary Files

File Name: Supplementary Data 1
Description: Differential interactions at 20kb resolution identified using diffHiC in TAMR vs. MCF7 cells at FDR< 0.05.

File Name: Supplementary Data 2
Description: Differential interactions at 20kb resolution identified using diffHiC in FASR vs. MCF7 cells at FDR< 0.05.

File Name: Supplementary Data 3
Description: Genes present at anchors of differential interactions identified using diffHiC in FASR vs. MCF7 cells and in TAMR vs. MCF7 cells.

File Name: Supplementary Data 4
Description: Genes with promoters located at anchors of differential interactions associated with endocrine-associated SNVs at lost CTCF sites.

File Name: Supplementary Data 5
Description: ReMap 2018 transcription factors in MCF7 cell enrichment at anchors of differential interactions.

File Name: Supplementary Data 6
Description: Sequencing, mapping and peak calling information for ChIP-seq datasets used in the study.
