# Supplementary material for: Epigenetic reprogramming at estrogen-receptor binding sites alters 3D chromatin landscape in endocrine-resistant breast cancer
Source: Nat Commun. 2020 Jan 16;11:320. doi: 10.1038/s41467-019-14098-x (PMC6965612; doi:10.1038/s41467-019-14098-x)
Supplement: Supplementary file 10 — Reporting Summary [file 41467_2019_14098_MOESM10_ESM.pdf]

nature research

Corresponding author(s): Susan Clark

Reporting Summary

Nature Research wishes to improve the reproducibility of the work that we publish. This form provides structure for consistency and transparency in reporting. For further information on Nature Research policies, see Authors & Referees and the Editorial Policy Checklist.

Statistical parameters

When statistical analyses are reported, confirm that the following items are present in the relevant location (e.g. figure legend, table legend, main text, or Methods section).

n/a	Confirmed	
☐	☒	The exact sample size (n) for each experimental group/condition, given as a discrete number and unit of measurement
☐	☒	An indication of whether measurements were taken from distinct samples or whether the same sample was measured repeatedly
☐	☒	The statistical test(s) used AND whether they are one- or two-sided<br>*Only common tests should be described solely by name; describe more complex techniques in the Methods section.*
☒	☐	A description of all covariates tested
☐	☒	A description of any assumptions or corrections, such as tests of normality and adjustment for multiple comparisons
☐	☒	A full description of the statistics including central tendency (e.g. means) or other basic estimates (e.g. regression coefficient) AND variation (e.g. standard deviation) or associated estimates of uncertainty (e.g. confidence intervals)
☐	☒	For null hypothesis testing, the test statistic (e.g. F, t, r) with confidence intervals, effect sizes, degrees of freedom and P value noted<br>*Give P values as exact values whenever suitable.*
☒	☐	For Bayesian analysis, information on the choice of priors and Markov chain Monte Carlo settings
☒	☐	For hierarchical and complex designs, identification of the appropriate level for tests and full reporting of outcomes
☒	☐	Estimates of effect sizes (e.g. Cohen's d, Pearson's r), indicating how they were calculated
☐	☒	Clearly defined error bars<br>*State explicitly what error bars represent (e.g. SD, SE, CI)*

Our web collection on statistics for biologists may be useful.

Software and code

Policy information about availability of computer code

Data collection

Hi-C
- HiC-Pro v2.9.0
- HiC-Pro/hicpro2juicebox.sh
- diffHiC v.1.15
- Bowtie2 v2.3.2
- “domain-caller” (Bing Ren: http://chromosome.sdsc.edu/mouse/hi-c/download.html)
- MATLAB vR2015b
- Homer v4.8

ChIP-seq
- NGSane v0.5.2.0
- Bowtie v1.1.0
- Masc2 v2.1.0
- diffBind v2.4.8
- chromHMM v1.17 (http://compbio.mit.edu/ChromHMM/)

WGS
- bwa-mem v0.7.9
- GATK v3.5

- QualiMap v2.1.3
- Mutect2 v3.8-0

WGBS
- Meth10X (https://github.com/luuloi/Meth10X)
- Bpipe v0.9.9.2
- Trim Galore v0.2.8
- Bwa-meth v0.20
- bwa v0.7.13
- Picard v2.3.0
- QualiMap v2.1.3
- MethylDackel (https://github.com/dpryan79/MethylDackel)
- Biscuit (https://github.com/zwdzwd/biscuit)
- Samtools v1.2

RNA-seq MCF7 vs. TAMR and MCF7 vs. FASR
- Trim Galore v0.11.2
- STAR v2.4.0j
- edgeR v3.18.1

Motif analysis
- Homer v4.7 findMotifsGenome.pl

Data analysis

All analysis was performed using open source software. All software code used to analyze the data for this study is publicly available as described in the methods section. All software used is published and/or in the public domain. All pipelines and R scripts used in the study are available at https://github.com/JoannaAch/MS_2019.

R software and packages: R v3.2.3, GenomicRanges v1.22.4, GenomicAlignments v1.6.3, rtracklayer v1.30.4, edgeR v3.12.1 limma v3.26.9, Repitools v1.16.0, GenomicFeatures v1.22.13, genomation v1.2.2, BSgenome.Hsapiens.UCSC.hg38_1.4.1, data.table v1.9.6, fpc v2.1-11, preprocessCore v1.32.0, ggthemes v3.0.3, reshape2 v1.4.1, ggplot2 v2.2.1, Rsamtools_1.28.0, DiffBind_2.4.8, genomation_1.8.0, diffHic_1.15.2

Other software: chromHMM v1.10, IGV v2.3.32, JuiceBox v1.6.2, deepTools2, Genomic association tester (GAT) v1.0, ngs.plot (https://github.com/shenlab-sinai/ngsplot), bedtools v2.25.0

For manuscripts utilizing custom algorithms or software that are central to the research but not yet described in published literature, software must be made available to editors/reviewers upon request. We strongly encourage code deposition in a community repository (e.g. GitHub). See the Nature Research guidelines for submitting code & software for further information.

Data

Policy information about availability of data

All manuscripts must include a data availability statement. This statement should provide the following information, where applicable:

- Accession codes, unique identifiers, or web links for publicly available datasets
- A list of figures that have associated raw data
- A description of any restrictions on data availability

All datasets used in this study are summarized in Supplementary Table 9. Raw and processed Hi-C, ChIP-seq, WGBS, WGS, RNA-seq and ChIP-seq data that support the findings of this study have been deposited in the NCBI Gene Expression Omnibus (GEO) with the primary accession code GSE118716 [https://www.ncbi.nlm.nih.gov/geo/query/acc.cgi?acc=GSE118716] and GSE130916 [https://www.ncbi.nlm.nih.gov/geo/query/acc.cgi?acc=GSE130916].

Field-specific reporting

Please select the best fit for your research. If you are not sure, read the appropriate sections before making your selection.

☒ Life sciences ☐ Behavioural & social sciences ☐ Ecological, evolutionary & environmental sciences

For a reference copy of the document with all sections, see nature.com/authors/policies/ReportingSummary-flat.pdf

Life sciences study design

All studies must disclose on these points even when the disclosure is negative.

Sample size

For Hi-C, RNA-seq, WGBS, WGS and ChIP-seq experiments two different endocrine resistant cell lines were used (tamoxifen-resistant and fulvestrant-resistant) to account for biological variability between datasets. For Hi-C and RNA-seq experiments were performed in triplicates to assess statistical significance.

Data exclusions

No data was excluded from analysis.

Replication

Hi-C experiments were performed in triplicates and reproducibility between replicates was verified using HiCRep (Yang T (2018)). RNA-seq

Replication	experiments were performed in triplicates. DNA methylation changes were replicated using patient's tumour samples. SNVs identified from WGS data were validated using dbSNP database as part of the Mutect2 pipeline. All findings were reproducible and instances of variability are discussed in the text.
Randomization	Randomization was not applicable to this study. All patient's included in the study received endocrine therapy.
Blinding	All patient tumour specimens were coded and blinded to the individuals running the assays. Investigators were not blinded during the process of computational analysis of Hi-C, RNA-seq, WGBS or WGS data.

Reporting for specific materials, systems and methods

Materials & experimental systems

n/a	Involved in the study	
☒	☐	Unique biological materials
☐	☒	Antibodies
☐	☒	Eukaryotic cell lines
☒	☐	Palaeontology
☒	☐	Animals and other organisms
☐	☒	Human research participants

Methods

n/a	Involved in the study	
☐	☒	ChIP-seq
☒	☐	Flow cytometry
☒	☐	MRI-based neuroimaging

Antibodies

Antibodies used	Antibodies used were H3K4me3 (Active Motif #39159), H3K4me1 (Active Motif, #39297), H3K27ac (Active Motif, #39133), H2AZac (Abcam, #ab18262), H3K27me3 (Millipore, #07-449) and CTCF (Millipore #07-729).
Validation	The following antibodies are validated for ChIP-seq in the Antibody Validation Database (Egelhofer et al. (2010)): H3K4me3 (Active Motif #39159), H3K4me1 (Active Motif, #39297), H3K27ac (Active Motif, #39133) and H3K27me3 (Millipore, #07-449). CTCF antibody (Millipore #07-729) has been used in multiple papers, including Taberlay et al. (2014). H2AZac (Abcam, #ab18262) is verified in Valdes-Mora et al. (2017).

Eukaryotic cell lines

Policy information about cell lines

Cell line source(s)	Parental MCF7 breast cancer cells and endocrine-resistant TAMR and FASR cells were kindly given to our laboratory by Dr Julia Gee (Cardiff University, UK).
Authentication	All cell lines were authenticated by short-tandem repeat profiling (CellBank Australia, Westmead, NSW, Australia) and cultured for <6 months after authentication.
Mycoplasma contamination	All cell lines used in-house tested negative for mycoplasma using the MycoAlert Mycoplasma Detection Kit (Lonza, #LT07-318).
Commonly misidentified lines (See ICLAC register)	No cell lines from the ICLAC register were used.

Human research participants

Policy information about studies involving human research participants

Population characteristics	Matched primary and metastatic tumour samples (n = 5) have been collected via needle microdissection between 1993 and 2013 by Dr Dave S. Hoon (John Wayne Cancer Institute, Los Angeles, USA). All patients were female, had ER-positive tumours and were treated with endocrine therapy. Patient's age at diagnosis was between 44 - 62 years.
Recruitment	Patient's were recruited into the study by their primary oncologies Dr Hoon.

ChIP-seq

Data deposition

☒ Confirm that both raw and final processed data have been deposited in a public database such as GEO.

☒ Confirm that you have deposited or provided access to graph files (e.g. BED files) for the called peaks.

Data access links
May remain private before publication.

Reviewer access link: https://www.ncbi.nlm.nih.gov/geo/query/acc.cgi?acc=GSE118716

Files in database submission

GSM3336876 MCF7_H2AZac_ChIP-seq
GSM3336877 TAMR_H2AZac_ChIP-seq
GSM3336878 FASR_H2AZac_ChIP-seq
GSM3336879 TAMR_H3K4me3_ChIP-seq
GSM3336880 FASR_H3K4me3_ChIP-seq
GSM3336881 TAMR_H3K4me1_ChIP-seq
GSM3336882 FASR_H3K4me1_ChIP-seq
GSM3336883 TAMR_H3K27me3_ChIP-seq
GSM3336884 TAMR_H3K27ac_ChIP-seq
GSM3336885 FASR_H3K27ac_ChIP-seq
GSM3336886 TAMR_CTCF_ChIP-seq
GSM3336887 FASR_CTCF_ChIP-seq
GSM3336888 TAMR_input_ChIP-seq
GSM3336889 FASR_input_ChIP-seq

Genome browser session
(e.g. UCSC)

Hi-C JuiceBox browser files are provided in the GEO submission. These files can be imported directly into JuiceBox and WashU Browser. ChIP-seq data generated in this paper has been used for the identification of chromatin states using chromHMM. ChromHMM segmentation files are provided in the GEO submission.

Methodology

Replicates

Two endocrine resistant cell lines were used in the study (TAMR and FASR). ChIP-seq experiments generated in this study were analysed for chromatin state identification using chromHMM. ChromHMM integrates multiple histone modification profiles to identify each state. ChromHMM emission profiles can be provided upon request. Public ER and CTCF ChIP-seq were performed in triplicates (MCF7 cells) and duplicates (TAMR and FASR cells).

Sequencing depth

Cell Line ChIP Total Reads Unique Length
MCF7_H2AZac_ChIP-seq 40,335,571 34,935,555 50bp SE
TAMR_H2AZac_ChIP-seq 39,693,078 34,612,970 50bp SE
FASR_H2AZac_ChIP-seq 38,049,204 33,027,138 50bp SE
TAMR_H3K4me3_ChIP-seq 24,853,306 22,230,53450bp SE
FASR_H3K4me3_ChIP-seq 48,137,289 42,058,596 50bp SE
TAMR_H3K4me1_ChIP-seq 34,719,315 30,338,216 50bp SE
FASR_H3K4me1_ChIP-seq 44,539,909 38,918,687 50bp SE
TAMR_H3K27me3_ChIP-seq 28,473,456 22,805,667 50bp SE
TAMR_H3K27ac_ChIP-seq 35,932,981 31,659,387 50bp SE
FASR_H3K27ac_ChIP-seq 33,675,069 29,398,564 50bp SE
TAMR_CTCF_ChIP-seq 50,187,808 43,665,211 50bp SE
FASR_CTCF_ChIP-seq 55,023,455 47,236,083 50bp SE
TAMR_input_ChIP-seq 18,912,303 12,619,645 50bp SE
FASR_input_ChIP-seq 11,811,049 7,508,650 50bp SE

Antibodies

Antibodies used were H3K4me3 (Active Motif #39159), H3K4me1 (Active Motif, #39297), H3K27ac (Active Motif, #39133), H2AZac (Abcam, #ab18262), H3K27me3 (Millipore, #07-449) and CTCF (Millipore #07-729).

Peak calling parameters

Peaks were called with macs2 software (2.1.0) under the default parameters (band width = 300, model fold = [5, 50], qvalue cutoff = 5.00e-02)

Data quality

ChIP-seq libraries were validated by qPCR (n=3). All peaks are below the Macs2 FDR cut off.

Software

All ChIP-seq raw datasets were mapped and processed through the NGSane framework (v.0.5.2.0)43. Reads were mapped to genome build hg38/GRCh38 with bowtie v.1.1.0 and mismatched (>3 mismatched bases), multiple mapping and duplicate reads were excluded from downstream analysis.
